# Rupture of the Gastrocnemius, &c.

**Published:** 1880-07

**Authors:** Geo. H. Berns


### ABSTRACT AND SUMMARY.

### CASE DEPARTMENT.

### I.—RUPTURE OF THE GASTROCNEMIUS AND TENDONS OF THE FLEXOR PEDIS PERFORATUS MUSCLES.

As this lesion is considered of very rare occurrence in the horse, it may prove interesting to the profession if I report three cases which came under my observation within the last eight months; all of which presented a peculiar train of symptoms.

*Case No. 1.*—On September 12th, 1879, I was called to see an aged bay horse, owned by Messrs. Howard & Fuller, of this city. The attendant informed me that the horse had slipped and had fallen in backing a heavy load, that he had considerable difficulty in getting up, and after he got up, evinced great lameness in the off hind leg.

When I saw him some three or four hours after the accident, he was standing with the off hind leg flexed, and further advanced in under his body than usual. He showed symptoms of great pain, breathing hurriedly and was covered with perspiration. While at rest the summit of the os calcis of the right hind leg appeared to be some eight or ten inches lower down than that of the left one, giving the thigh a peculiar elongated appearance. When forced to throw any weight upon affected limb, the metatarsus would be flexed upon the tibia to such an extent, as to form almost an acute angle.

The strong tendons of the gastrocnemius and flexor pedis perforatus muscles (tendon of Achilles) were slightly relaxed when the leg was at rest. No heat, no swelling, no pain on pressure could be detected anywhere, and there was no knuckling over at the fetlock. The horse walked moderately well, but every time any weight was thrown upon the limb, the point of the hock would drop to within six or eight inches of the ground, and the parts below the hock would be flexed upon the tibia.

DIAGNOSIS.—Rup'ure of the gastrocnemius muscles. I made an attempt to keep the leg in a proper position by splints, and the use of plaster Paris, but found it impossible to retain it in this position long enough to adjust the necessary bandages, etc. The patient being old, treatment was abandoned, and the horse ordered destroyed.

Autopsy made immediately after death revealed a complete rupture of muscular fibres of the fleshy belly of the gastrocnemius, below the union of its two heads, also laceration of the flexor pedis perforatus muscle in the same region. The fibres above and below the rupture had contracted, and a cavity some six inches in diameter and about four inches deep, was found filled with coagulated blood.

*Case No. 2.*—This was a gray gelding, about ten years old and in good condition, the property of Mr. Rath, of Court Street, Brooklyn. I was called to see this horse April 18th, 1880, and found him standing very much in the same position as Howard & Fuller's horse. Point of the hock very much lower than its fellow, extreme flexion of the metatarsus, and dropping of the os calcis still lower on weight being thrown upon the limb. The horse so lame that he would hop on three legs when forced to move. The hock joint, particularly in the region of the calcaneo-cuboid ligament, the summit of the os calcis, and immediately above it was greatly swollen, hot, and very sensitive to the touch.

The owner informed me that the horse had been standing in the stable some two or three weeks, suffering from acute erythema of the pastern. On entering the stable one morning, three days previous to my seeing him, he found the patient in the condition above described. I ordered cold water bathing and a cooling sedative lotion to be applied to the swollen parts.

Saw my patient again two days later in company with Dr. Housman, and found that the swelling and tenderness had almost subsided, but the peculiar elongated condition of the thigh, and lowering of the point of the hock was more marked. An examination of the parts now revealed that the cartilaginous cap of the tendon of the flexor pedis perforatus muscle, had slipped from its place, the summit of the os calcis, to about an inch above it, and could be distinctly felt. The tendon of Achilles, above the place where the cap was felt, was relaxed.

Deeming treatment inadvisable, the horse was destroyed, and the autopsy revealed a rupture of the tendon of the flexor pedis perforatus muscle, just below the summit of the os calcis, and the tendon of the gastrocnemius had been torn from its insertion.

*Case No. 3.*—Behrens Bros., of Concord and Pearl Streets, Brooklyn, sent for me April 29th, 1880, to see a lame horse. I learned that the horse had worked to a grocer's wagon all the morning, was put into the stable at one o'clock P. M., apparently in the best of health, and a half an hour later, when the clerk went in to feed him, he was found in the condition in which I first saw him.

He was standing in the same peculiar position as the other two horses. The affected limb well advanced in under the body, thigh elongated, point of the hock six or eight inches from the floor. When any weight was thrown upon the limb, the same extreme flexion of the metatarsus, and the formation of an acute angle was as noticeable as in the other two cases. There was no swelling, heat or tenderness anywhere.

Diagnosed Rupture of Gastrocnemius and ordered the horse destroyed.

Autopsy revealed that the external head of the gastrocnemius had been torn from its attachment to the femur, taking a piece of that bone with it. The fleshy belly of its internal head was ruptured immediately above its union with the external.

GEO. H. BERNS, D. V. S.

